# The genome sequence of the Spotted Sulphur, *Emmelia trabealis* (Scopoli, 1763) (Lepidoptera: Noctuidae)

**DOI:** 10.12688/wellcomeopenres.25825.1

**Published:** 2026-02-16

**Authors:** Roger Vila, Ján Krupa, Charlotte J. Wright, Joana I. Meier, Mark L. Blaxter, Wellcome Sanger Institute Tree of Life Management, Samples and Laboratory team, Wellcome Sanger Institute Scientific Operations: Sequencing Operations, Wellcome Sanger Institute Tree of Life Core Informatics team, Tree of Life Core Informatics collective, Project Psyche Community

**Affiliations:** 1 Institut de Biologia Evolutiva (CSIC-UPF), Barcelona, Catalonia, Spain; 2 Biology Centre of the Czech Academy of Sciences, Institute of Entomology, České Budějovice, Czech Republic; 3 Department of Zoology, Faculty of Science, University of South Bohemia, České Budějovice, Czech Republic; 4 Tree of Life Programme, Wellcome Sanger Institute, Hinxton, England, UK

**Keywords:** Emmelia trabealis; Spotted Sulphur; genome sequence; chromosomal; Lepidoptera

## Abstract

We present a genome assembly from a female specimen of *Emmelia trabealis* (Spotted Sulphur; Arthropoda; Insecta; Lepidoptera; Noctuidae). The assembly contains two haplotypes with total lengths of 550.30 megabases and 512.33 megabases. Most of haplotype 1 (98.62%) is scaffolded into 32 chromosomal pseudomolecules, including the W and Z sex chromosomes. Haplotype 2 was assembled to scaffold level. The mitochondrial genome has also been assembled, with a length of 15.66 kilobases. This work is part of Project Psyche, a collaborative programme generating genomes for European butterflies and moths.

## Species taxonomy

Eukaryota; Opisthokonta; Metazoa; Eumetazoa; Bilateria; Protostomia; Ecdysozoa; Panarthropoda; Arthropoda; Mandibulata; Pancrustacea; Hexapoda; Insecta; Dicondylia; Pterygota; Neoptera; Endopterygota; Amphiesmenoptera; Lepidoptera; Glossata; Neolepidoptera; Heteroneura; Ditrysia; Obtectomera; Noctuoidea; Noctuidae; Acontiinae; *Emmelia*; *Emmelia trabealis* (Scopoli, 1763) (NCBI:txid938230)

## Background

**Figure 1.**
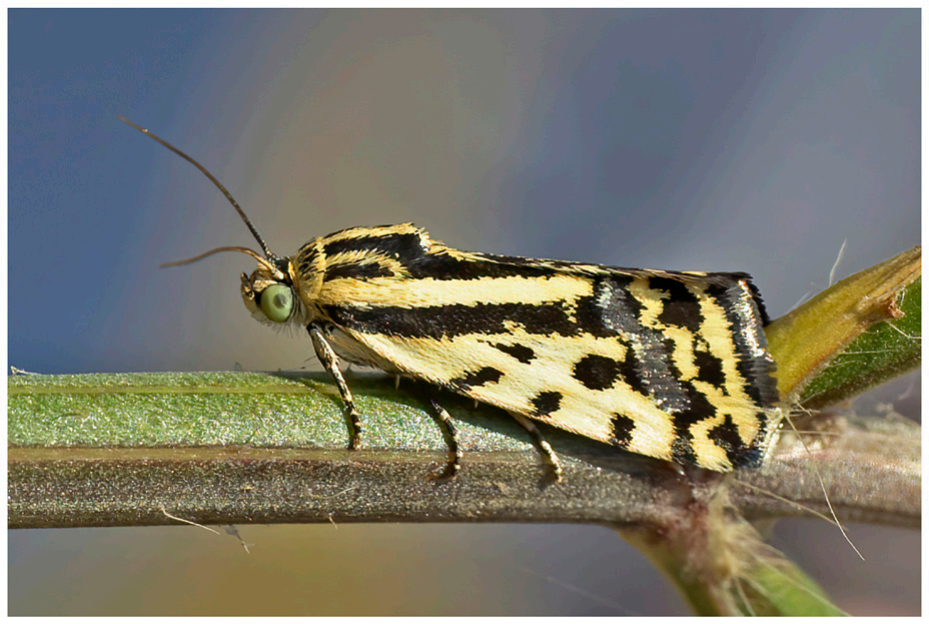
Image of *Emmelia trabealis* by Charles J. Sharpe.

The Spotted Sulphur ( Figure 1) is a noctuid moth belonging to the tribe Acontiini. Its distribution extends from north Africa, including Madeira and the Canary Islands, through Europe to Japan ( Macek *et al.*, 2008). In other parts of its range, especially in north-western Europe, a significant decline has been recorded due to the development of intensive farming. The species has been considered extinct in Britain since 1960 ( Macek *et al.*, 2008; Waring *et al.*, 2003). *E. trabealis* inhabits both lowland areas and mountainous regions up to 2 000 m asl ( Fibiger *et al.*, 2009; Macek *et al.*, 2008).

The wingspan of this species ranges from 20 to 22 mm. The forewings are highly variable, usually with black lines and dots on a yellow or off-white background, varying from entirely yellow to entirely black specimens; the hindwings are reddish-brown ( Fibiger *et al.*, 2009; Waring *et al.*, 2003).

Adults usually fly during the day in two, occasionally three, overlapping generations per year, typically from May to July and again from August to September, depending on latitude. *Emmelia trabealis* prefers open, dry, sandy habitats ( Macek *et al.*, 2008).

The larva is monophagous, feeding on the flowers of *Convolvulus arvensis* and occurs in two generations, from June to July and from August to September. It is nocturnal, with younger instars hiding during the day in the flowers of *Convolvulus arvensis* and older instars among low-growing vegetation. It pupates in a cocoon on the ground, where it can overwinter. Adults may emerge the following spring or in the second year after pupation ( Macek *et al.*, 2008).

We present a chromosome-level, haplotype-resolved genome sequence for *E. trabealis*, sequenced as part of Project Psyche. The sequence data were derived from a female specimen ( Figure 1) collected from Cardedeu, Vallès Oriental, Barcelona, Catalonia, Spain.

## Methods

### Sample acquisition

The specimen used for genome sequencing was an adult female *Emmelia trabealis* (specimen ID SAN28000261, ToLID ilEmmTrab1), collected from Cardedeu, Vallès Oriental, Barcelona, Catalonia, Spain (latitude 41.6527, longitude 2.3546) on 2023-09-18. The specimen was collected and identified by Roger Vila (Institut de Biologia Evolutiva).

### Nucleic acid extraction

Protocols for high molecular weight (HMW) DNA extraction developed at the Wellcome Sanger Institute (WSI) Tree of Life Core Laboratory are available on protocols.io ( Howard *et al.*, 2025). The ilEmmTrab1 sample was weighed and triaged to determine the appropriate extraction protocol. Tissue from the whole organism was homogenised by powermashing using a PowerMasher II tissue disruptor.

HMW DNA was extracted in the WSI Scientific Operations core using the Automated MagAttract v2 protocol. DNA was sheared into an average fragment size of 12–20 kb following the Megaruptor®3 for LI PacBio protocol. Sheared DNA was purified by automated SPRI (solid-phase reversible immobilisation). The concentration of the sheared and purified DNA was assessed using a Nanodrop spectrophotometer and Qubit Fluorometer using the Qubit dsDNA High Sensitivity Assay kit. Fragment size distribution was evaluated by running the sample on the FemtoPulse system. For this sample, the final post-shearing DNA had a Qubit concentration of 37.37 ng/μL and a yield of 1 756.39 ng, with a fragment size of 15.4 kb.

### PacBio HiFi library preparation and sequencing

Library preparation and sequencing were performed at the WSI Scientific Operations core. Libraries were prepared using the SMRTbell Prep Kit 3.0 (Pacific Biosciences, California, USA), according to the manufacturer’s instructions. The kit includes reagents for end repair/A-tailing, adapter ligation, post-ligation SMRTbell bead clean-up, and nuclease treatment. Size selection and clean-up were performed using diluted AMPure PB beads (Pacific Biosciences). DNA concentration was quantified using a Qubit Fluorometer v4.0 (ThermoFisher Scientific) and the Qubit 1X dsDNA HS assay kit. Final library fragment size was assessed with the Agilent Femto Pulse Automated Pulsed Field CE Instrument (Agilent Technologies) using the gDNA 55 kb BAC analysis kit. The sample was sequenced on a Revio instrument (Pacific Biosciences). The prepared library was normalised to 2 nM, and 15 μL was used for making complexes. Primers were annealed and polymerases bound to generate circularised complexes, following the manufacturer’s instructions. Complexes were purified using 1.2X SMRTbell beads, then diluted to the Revio loading concentration (200–300 pM) and spiked with a Revio sequencing internal control. The sample was sequenced on a Revio 25M SMRT cell. The SMRT Link software (Pacific Biosciences), a web-based workflow manager, was used to configure and monitor the run and to carry out primary and secondary data analysis.

Specimen details, sequencing platforms, and data yields are summarised in Table 1.

**Table 1.** Specimen and sequencing data for BioProject PRJEB80925.

Platform	PacBio HiFi	Hi-C
**ToLID**	ilEmmTrab1	ilEmmTrab1
**Specimen ID**	SAN28000261	SAN28000261
**BioSample (source individual)**	SAMEA115768767	SAMEA115768767
**BioSample (tissue)**	SAMEA115768845	SAMEA115768845
**Tissue**	whole organism	whole organism
**Instrument**	Revio	Illumina NovaSeq X
**Run accessions**	ERR13800480	ERR13802624
**Read count total**	6.05 million	656.38 million
**Base count total**	67.06 Gb	99.11 Gb

### Hi-C

***Sample preparation and crosslinking***

The Hi-C sample was prepared from 20–50 mg of frozen tissue from the whole organism of the ilEmmTrab1 sample using the Arima-HiC v2 kit (Arima Genomics). Following the manufacturer’s instructions, tissue was fixed and DNA crosslinked using TC buffer to a final formaldehyde concentration of 2%. The tissue was homogenised using the Diagnocine Power Masher-II. Crosslinked DNA was digested with a restriction enzyme master mix, biotinylated, and ligated. Clean-up was performed with SPRISelect beads before library preparation. DNA concentration was measured with the Qubit Fluorometer (Thermo Fisher Scientific) and Qubit HS Assay Kit. The biotinylation percentage was estimated using the Arima-HiC v2 QC beads.

***Hi-C library preparation and sequencing***

Biotinylated DNA constructs were fragmented using a Covaris E220 sonicator and size selected to 400–600 bp using SPRISelect beads. DNA was enriched with Arima-HiC v2 kit Enrichment beads. End repair, A-tailing, and adapter ligation were carried out with the NEBNext Ultra II DNA Library Prep Kit (New England Biolabs), following a modified protocol where library preparation occurs while DNA remains bound to the Enrichment beads. Library amplification was performed using KAPA HiFi HotStart mix and a custom Unique Dual Index (UDI) barcode set (Integrated DNA Technologies). Depending on sample concentration and biotinylation percentage determined at the crosslinking stage, libraries were amplified with 10–16 PCR cycles. Post-PCR clean-up was performed with SPRISelect beads. Libraries were quantified using the AccuClear Ultra High Sensitivity dsDNA Standards Assay Kit (Biotium) and a FLUOstar Omega plate reader (BMG Labtech). Prior to sequencing, libraries were normalised to 10 ng/μL. Normalised libraries were quantified again to create equimolar and/or weighted 2.8 nM pools. Pool concentrations were checked using the Agilent 4200 TapeStation (Agilent) with High Sensitivity D500 reagents before sequencing. Sequencing was performed using paired-end 150 bp reads on the Illumina NovaSeq X.

Specimen details, sequencing platforms, and data yields are summarised in Table 1.

### Genome assembly

Prior to assembly of the PacBio HiFi reads, a database of *k*-mer counts ( *k* = 31) was generated from the filtered reads using FastK. GenomeScope2 ( Ranallo-Benavidez *et al.*, 2020) was used to analyse the *k*-mer frequency distributions, providing estimates of genome size, heterozygosity, and repeat content.

The HiFi reads were assembled using Hifiasm in Hi-C phasing mode ( Cheng *et al.*, 2021; Cheng *et al.*, 2022), producing two haplotypes. Hi-C reads ( Rao *et al.*, 2014) were mapped to the primary contigs using bwa-mem2 ( Vasimuddin *et al.*, 2019). Contigs were further scaffolded with Hi-C data in YaHS ( Zhou *et al.*, 2023), using the --break option for handling potential misassemblies. The scaffolded assemblies were evaluated using Gfastats ( Formenti *et al.*, 2022), BUSCO ( Manni *et al.*, 2021) and MERQURY.FK ( Rhie *et al.*, 2020).

The mitochondrial genome was assembled using MitoHiFi ( Uliano-Silva *et al.*, 2023), which runs MitoFinder ( Allio *et al.*, 2020) and uses these annotations to select the final mitochondrial contig and to ensure the general quality of the sequence.

### Assembly curation

The assembly was decontaminated using the Assembly Screen for Cobionts and Contaminants ( ASCC) pipeline. TreeVal was used to generate the flat files and maps for use in curation. Manual curation was conducted primarily in PretextView and HiGlass ( Kerpedjiev *et al.*, 2018). Scaffolds were visually inspected and corrected as described by Howe *et al*. (2021). Manual corrections included 29 breaks, 77 joins, and removal of 33 haplotypic duplications. This reduced the scaffold count by 16.7%, increased scaffold N50 by 0.5%, and reduced the total assembly length by 1.0%. The curation process is described at https://gitlab.com/wtsi-grit/rapid-curation. PretextSnapshot was used to generate a Hi-C contact map of the final assembly.

### Assembly quality assessment

The Merqury.FK tool ( Rhie *et al.*, 2020), run in a Singularity container ( Kurtzer *et al.*, 2017), was used to evaluate *k*-mer completeness and assembly quality for both haplotypes using the *k*-mer database ( *k* = 31) computed prior to genome assembly. The analysis outputs included assembly QV scores and completeness statistics.

The genome was analysed using the BlobToolKit pipeline, a Nextflow ( Di Tommaso *et al.*, 2017) implementation of the earlier Snakemake version ( Challis *et al.*, 2020). The pipeline aligns PacBio reads using minimap2 ( Li, 2018) and SAMtools ( Danecek *et al.*, 2021) to generate coverage tracks. It runs BUSCO ( Manni *et al.*, 2021) using lineages identified from the NCBI Taxonomy ( Schoch *et al.*, 2020). For the three domain-level lineages, BUSCO genes are aligned to the UniProt Reference Proteomes database ( Bateman *et al.*, 2023) using DIAMOND blastp ( Buchfink *et al.*, 2021). The genome is divided into chunks based on the density of BUSCO genes from the closest taxonomic lineage, and each chunk is aligned to the UniProt Reference Proteomes database with DIAMOND blastx. Sequences without hits are chunked using seqtk and aligned to the NT database with blastn ( Altschul *et al.*, 1990). The BlobToolKit suite consolidates all outputs into a blobdir for visualisation. The BlobToolKit pipeline was developed using nf-core tooling ( Ewels *et al.*, 2020) and MultiQC ( Ewels *et al.*, 2016), with containerisation through Docker ( Merkel, 2014) and Singularity ( Kurtzer *et al.*, 2017).

We painted Merian elements, which represent the 32 ancestral linkage groups in Lepidoptera ( Wright *et al.*, 2024), along chromosomes using lep_busco_painter. Painting used BUSCO gene locations from the lepidoptera_odb10 set ( Kriventseva *et al.*, 2019) and chromosome lengths from NCBI Datasets. Each complete BUSCO (including both single-copy and duplicated BUSCOs) was assigned to a Merian element using a reference database. Positions were coloured and plotted along chromosomes drawn to scale.

## Genome sequence report

### Sequence data

**Figure 2.**
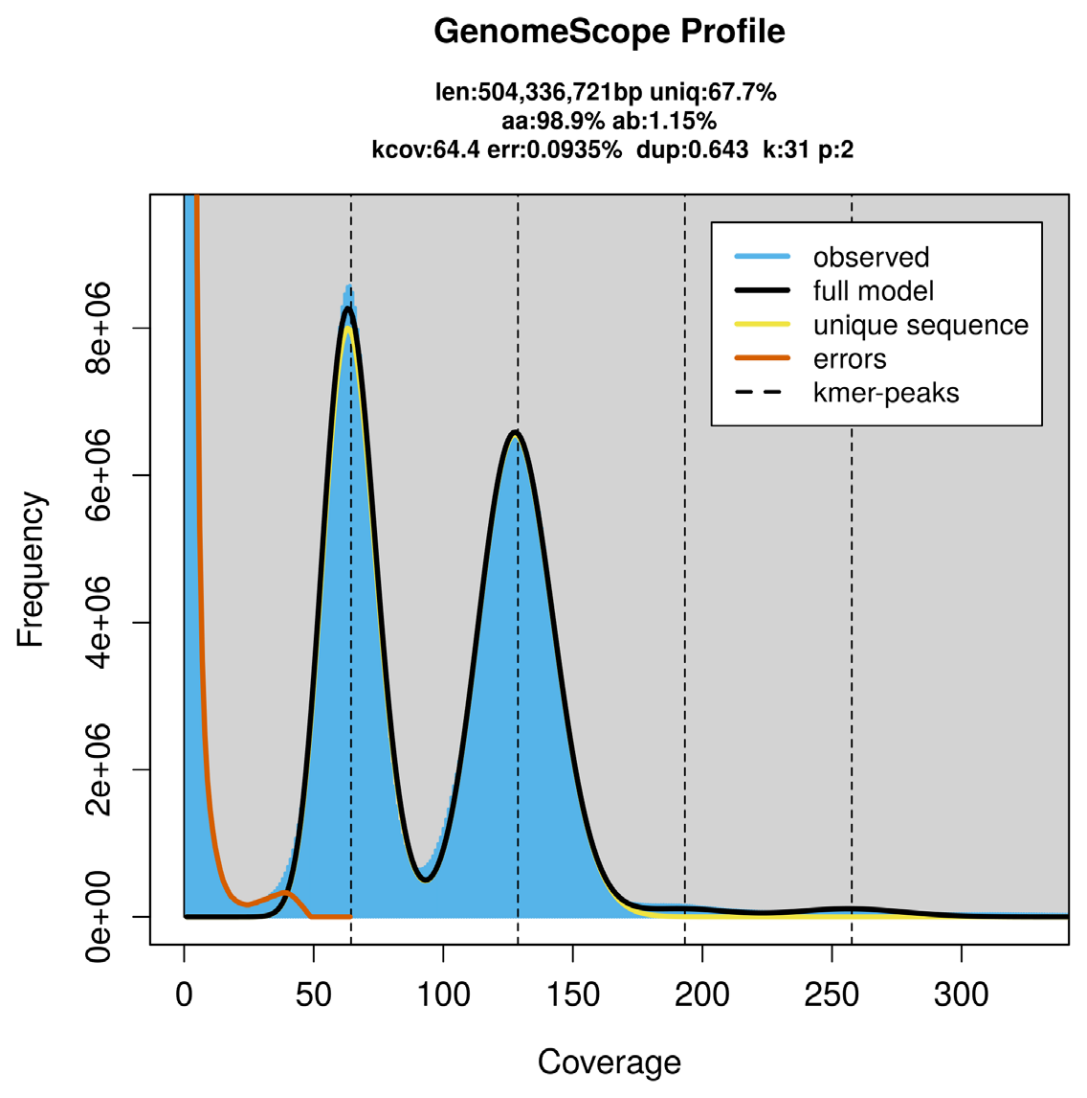
Frequency distribution of *k*-mers generated using GenomeScope2. The plot shows observed and modelled *k*-mer spectra, providing estimates of genome size, heterozygosity, and repeat content based on unassembled sequencing reads.

PacBio sequencing of the *Emmelia trabealis* specimen generated 67.06 Gb (gigabases) from 6.05 million reads, which were used to assemble the genome. GenomeScope2.0 analysis estimated the haploid genome size at 504.63 Mb, with a heterozygosity of 1.15% and repeat content of 32.34% ( Figure 2). These estimates guided expectations for the assembly. Based on the estimated genome size, the sequencing data provided approximately 129× coverage. Hi-C sequencing produced 99.11 Gb from 656.38 million reads, which were used to scaffold the assembly.

Table 1 summarises the specimen and sequencing details.

### Assembly statistics

The genome was assembled into two haplotypes using Hi-C phasing. Haplotype 1 was curated to chromosome level, while haplotype 2 was assembled to scaffold level. The final assembly has a total length of 550.30 Mb in 219 scaffolds, with 169 gaps, and a scaffold N50 of 17.14 Mb ( Table 2).

**Table 2.** Genome assembly statistics.

**Assembly name**	ilEmmTrab1.hap1.1	ilEmmTrab1.hap2.1
**Assembly accession**	GCA_964291695.1	GCA_964291385.1
**Assembly level**	chromosome	scaffold
**Span (Mb)**	550.30	512.33
**Number of chromosomes**	32	scaffold-level
**Number of contigs**	388	589
**Contig N50**	4.4 Mb	5.6 Mb
**Number of scaffolds**	219	436
**Scaffold N50**	17.14 Mb	16.64 Mb
**Longest scaffold length (Mb)**	25.99	-
**Sex chromosomes**	W and Z	-
**Organelles**	Mitochondrion: 15.66 kb	-

**Figure 3.**
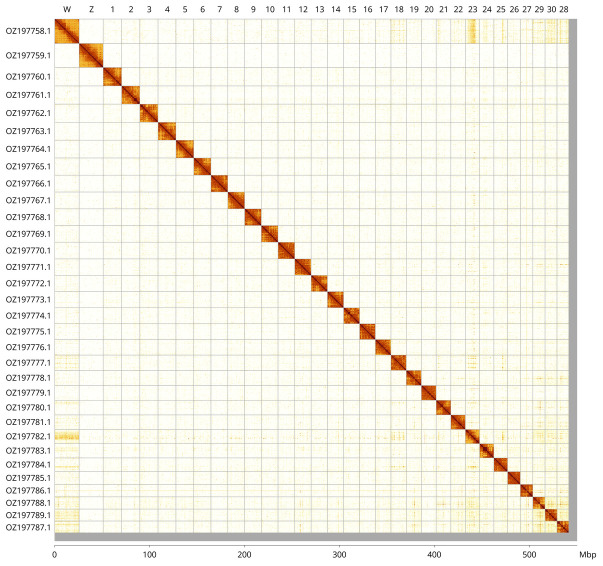
Hi-C contact map of the *Emmelia trabealis* genome assembly. Assembled chromosomes are shown in order of size and labelled along the axes, with a megabase scale shown below. The plot was generated using PretextSnapshot.

**Figure 4.**
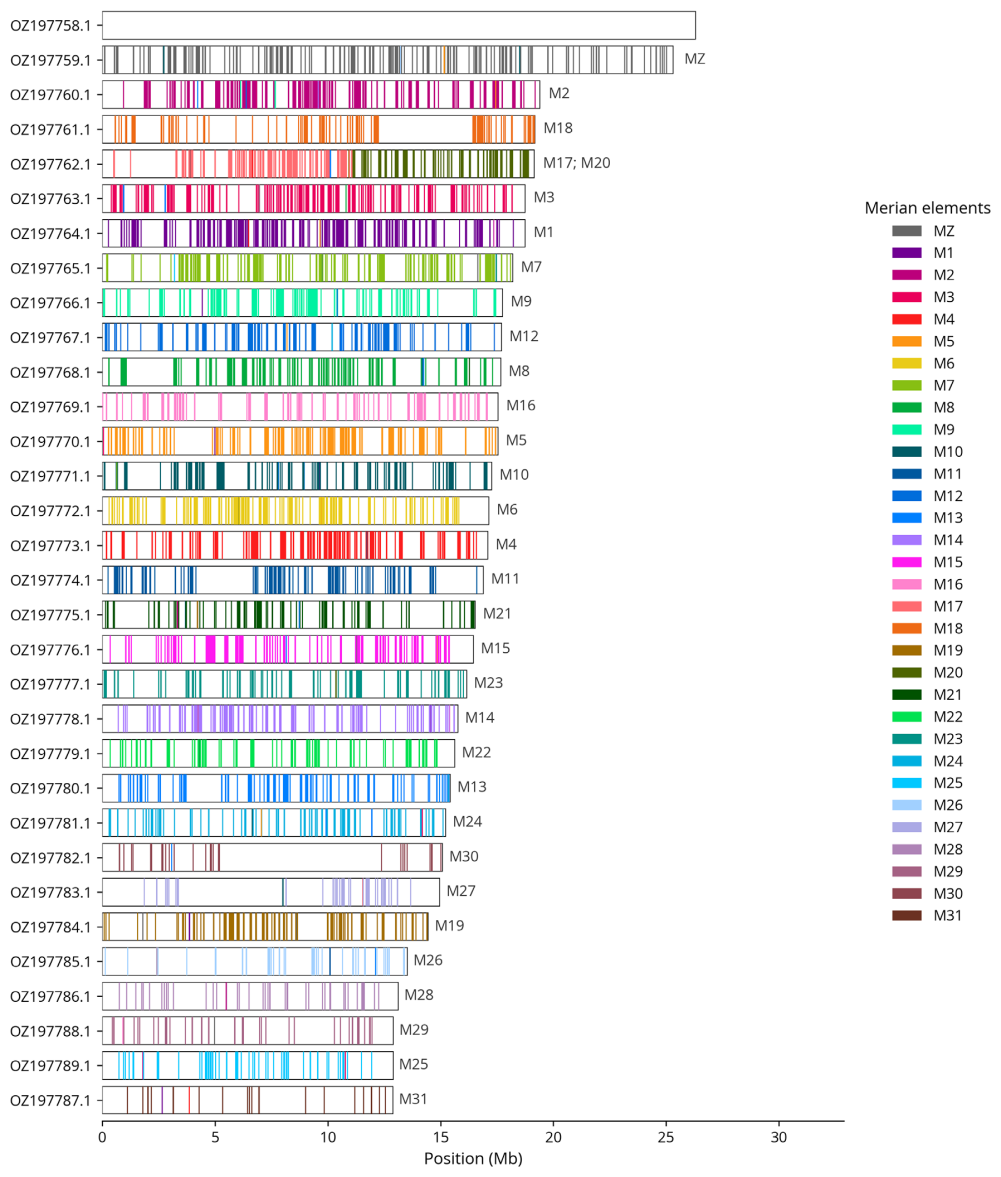
Merian elements painted across chromosomes in the ilEmmTrab1.hap1.1 assembly of *Emmelia trabealis*. Chromosomes are drawn to scale, with the positions of orthologues shown as coloured bars. Each orthologue is coloured by the Merian element that it belongs to. All orthologues which could be assigned to Merian elements are shown.

Most of the assembly sequence (98.62%) was assigned to 32 chromosomal-level scaffolds, representing 30 autosomes and the W and Z sex chromosomes. These chromosome-level scaffolds, confirmed by Hi-C data, are named according to size ( Figure 3; Table 3). Chromosome painting with Merian elements illustrates the distribution of orthologues along chromosomes and highlights patterns of chromosomal evolution relative to Lepidopteran ancestral linkage groups ( Figure 4). Chromosomes Z and W were assigned using read coverage data and alignment to *Acronicta aceris* (GCA_910591435.1) During curation, we observed inversions between haplotypes in the following chromosomes: Chromosome 19 from approximately 6.8–8.6 Mb, Chromosome 28 from approximately 7.8–10.3 Mb, and Chromosome 30 from approximately 4.3–6.2 Mb.

**Table 3.** Chromosomal pseudomolecules in the haplotype 1 genome assembly of *Emmelia trabealis* ilEmmTrab1.

INSDC accession	Molecule	Length (Mb)	GC%	Assigned Merian elements
OZ197760.1	1	19.41	36.50	M2
OZ197761.1	2	19.18	36	M18
OZ197762.1	3	19.16	37	M17;M20
OZ197763.1	4	18.75	37	M3
OZ197764.1	5	18.74	37	M1
OZ197765.1	6	18.20	37	M7
OZ197766.1	7	17.75	36.50	M9
OZ197767.1	8	17.70	36.50	M12
OZ197768.1	9	17.67	36.50	M8
OZ197769.1	10	17.55	37	M16
OZ197770.1	11	17.54	36.50	M5
OZ197771.1	12	17.26	36.50	M10
OZ197772.1	13	17.14	37	M6
OZ197773.1	14	17.09	37	M4
OZ197774.1	15	16.89	37	M11
OZ197775.1	16	16.54	36.50	M21
OZ197776.1	17	16.46	37.50	M15
OZ197777.1	18	16.15	37	M23
OZ197778.1	19	15.77	37	M14
OZ197779.1	20	15.63	36.50	M22
OZ197780.1	21	15.43	37	M13
OZ197781.1	22	15.23	37	M24
OZ197782.1	23	15.08	39	M30
OZ197783.1	24	14.96	36.50	M27
OZ197784.1	25	14.44	37.50	M19
OZ197785.1	26	13.53	37.50	M26
OZ197786.1	27	13.13	37.50	M28
OZ197787.1	28	12.89	38	M31
OZ197788.1	29	12.91	38	M29
OZ197789.1	30	12.90	38	M25
OZ197758.1	W	26.31	39	-
OZ197759.1	Z	25.31	36.50	MZ

The mitochondrial genome was also assembled (length 15.66 kb, OZ197790.1). This sequence is included as a contig in the multifasta file of the genome submission and as a standalone record.

### Assembly quality metrics

**Figure 5.**
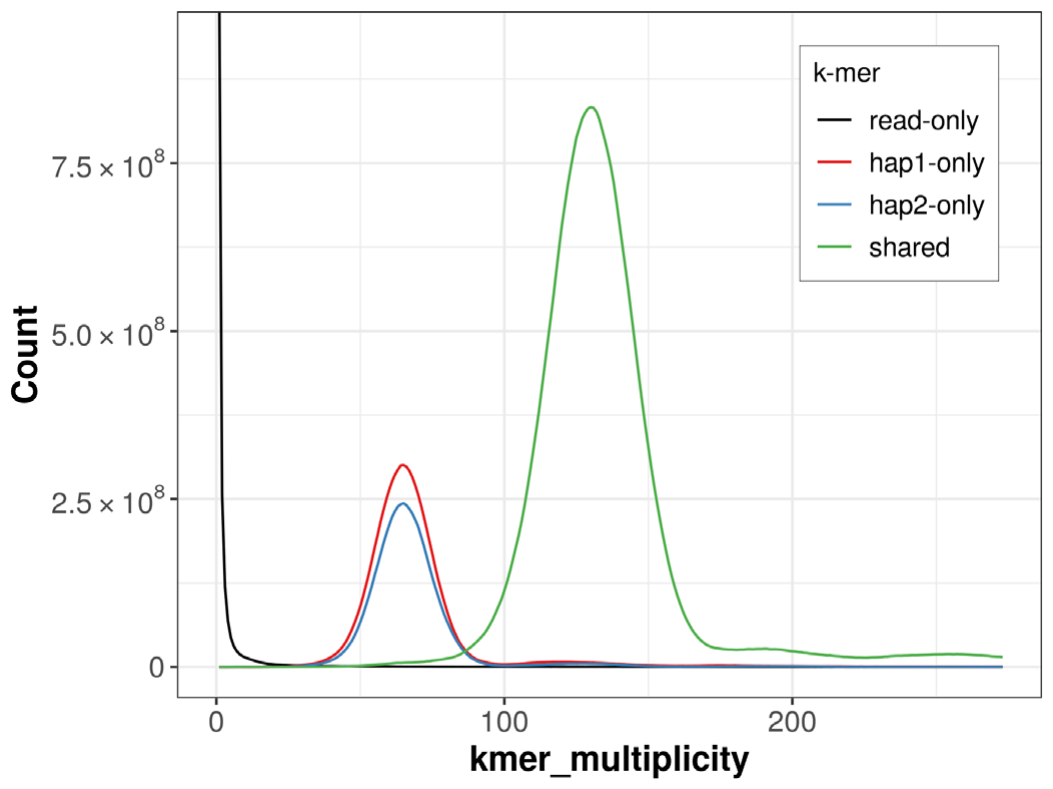
Evaluation of *k*-mer completeness using MerquryFK. This plot illustrates the recovery of *k*-mers from the original read data in the final assemblies. The horizontal axis represents *k*-mer multiplicity, and the vertical axis shows the number of *k*-mers. The black curve represents *k*-mers that appear in the reads but are not assembled. The green curve (the homozygous peak) corresponds to *k*-mers shared by both haplotypes and the red and blue curves (the heterozygous peaks) show *k*-mers found only in one of the haplotypes.

**Figure 6.**
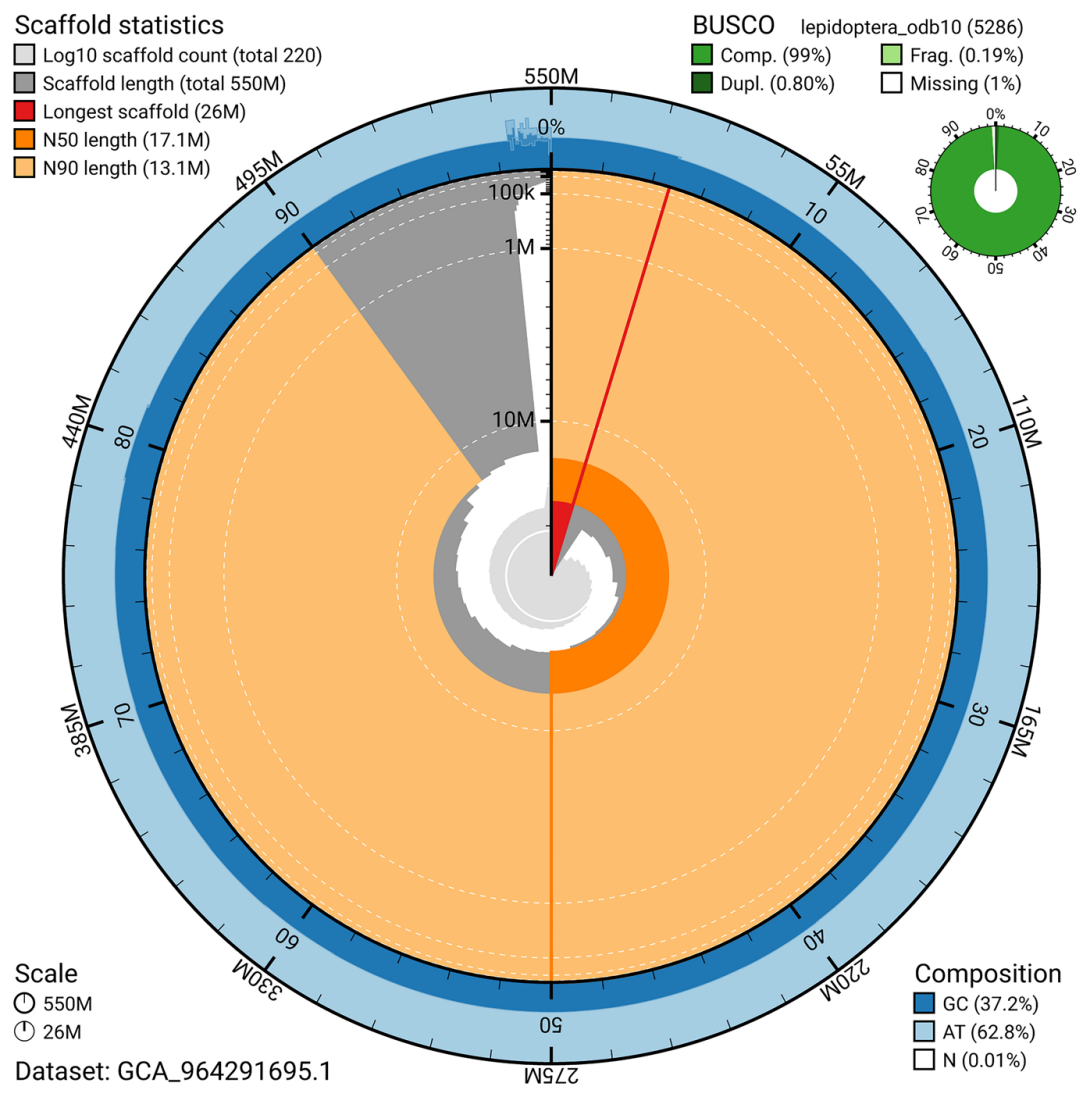
Assembly metrics for ilEmmTrab1.hap1.1. The BlobToolKit snail plot provides an overview of assembly metrics and BUSCO gene completeness. The circumference represents the length of the whole genome sequence, and the main plot is divided into 1,000 bins around the circumference. The outermost blue tracks display the distribution of GC, AT, and N percentages across the bins. Scaffolds are arranged clockwise from longest to shortest and are depicted in dark grey. The longest scaffold is indicated by the red arc, and the deeper orange and pale orange arcs represent the N50 and N90 lengths. A light grey spiral at the centre shows the cumulative scaffold count on a logarithmic scale. A summary of complete, fragmented, duplicated, and missing BUSCO genes in the set is presented at the top right. An interactive version of this figure can be accessed on the BlobToolKit viewer.

**Figure 7.**
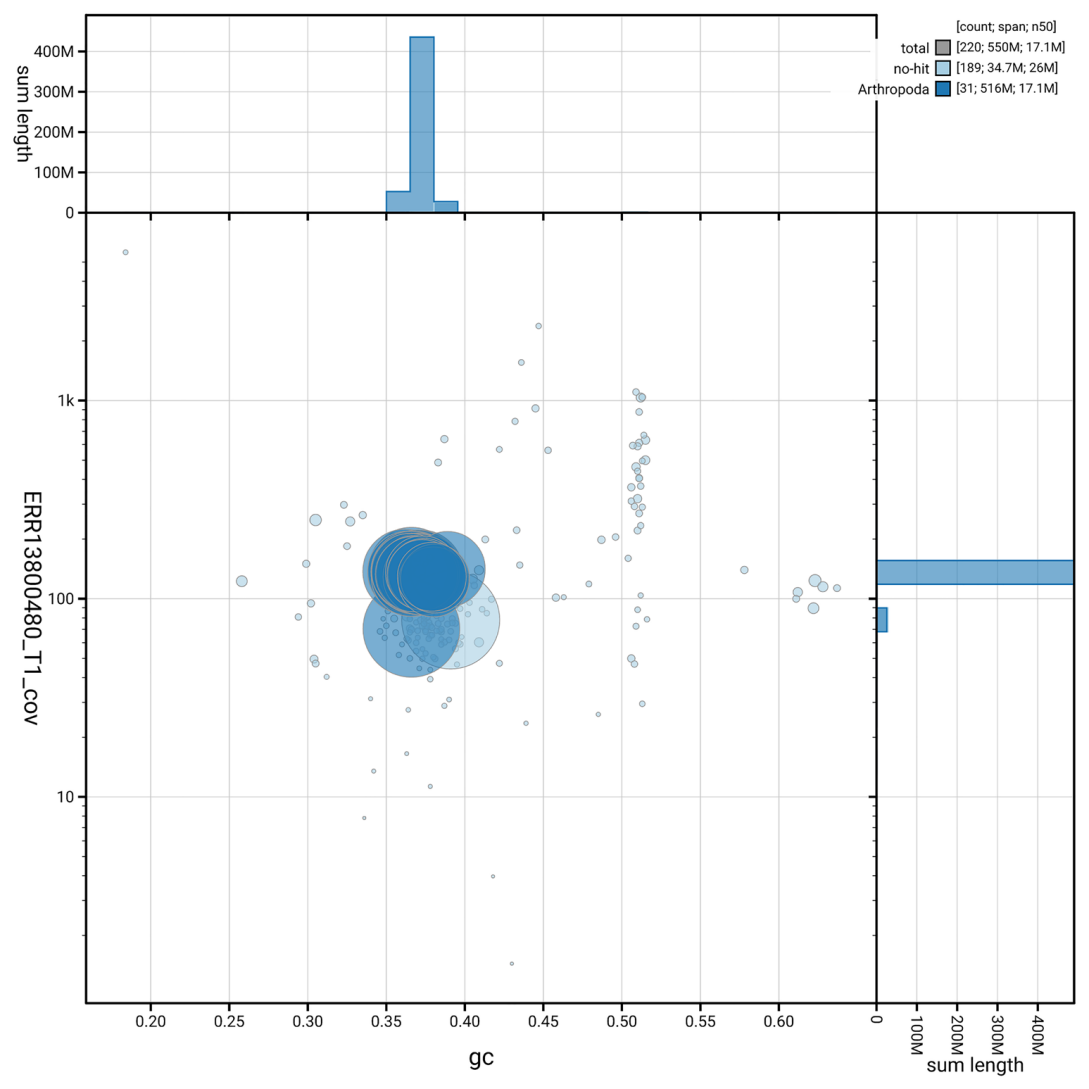
BlobToolKit GC-coverage plot for ilEmmTrab1.hap1.1. Blob plot showing sequence coverage (vertical axis) and GC content (horizontal axis). The circles represent scaffolds, with the size proportional to scaffold length and the colour representing phylum membership. The histograms along the axes display the total length of sequences distributed across different levels of coverage and GC content. An interactive version of this figure is available on the BlobToolKit viewer.

For haplotype 1, the estimated QV is 60.1, and for haplotype 2, 58.3. When the two haplotypes are combined, the assembly achieves an estimated QV of 59.1. The *k*-mer completeness is 80.24% for haplotype 1, 74.82% for haplotype 2, and 99.64% for the combined haplotypes ( Figure 5). BUSCO analysis using the lepidoptera_odb10 reference set ( *n* = 5 286) identified 99.0% of the expected gene set (single = 98.2%, duplicated = 0.8%) in haplotype 1. For haplotype 2, BUSCO analysis identified 94.7% of the expected gene set (single = 93.8%, duplicated = 0.9%). The snail plot in Figure 6 summarises the scaffold length distribution and other assembly statistics for haplotype 1. The blob plot in Figure 7 shows the distribution of scaffolds by GC proportion and coverage for haplotype 1.

Table 4 lists the assembly metric benchmarks adapted from Rhie *et al.* (2021) the Earth BioGenome Project Report on Assembly Standards September 2024. The EBP metric, calculated for the haplotype 1, is **6.C.Q60**, meeting the recommended reference standard.

**Table 4.** Earth Biogenome Project summary metrics for the *Emmelia trabealis* assembly.

Measure	Value	Benchmark
EBP summary (haplotype 1)	6.C.Q60	6.C.Q40
Contig N50 length	4.40 Mb	≥ 1 Mb
Scaffold N50 length	17.14 Mb	= chromosome N50
Consensus quality (QV)	Haplotype 1: 60.1; haplotype 2: 58.3; combined: 59.1	≥ 40
*k*-mer completeness	Haplotype 1: 80.24%; Haplotype 2: 74.82%; combined: 99.64%	≥ 95%
BUSCO	C:99.0% [S:98.2%; D:0.8%]; F:0.2%; M:0.8%; n:5 286	S > 90%; D < 5%
Percentage of assembly assigned to chromosomes	98.62%	≥ 90%

**Notes:** EBP summary uses log10(Contig N50); chromosome-level (C) or log10(Scaffold N50); Q (Merqury QV). BUSCO: C=complete; S=single-copy; D=duplicated; F=fragmented; M=missing; n=orthologues

### Wellcome Sanger Institute – Legal and Governance

The materials that have contributed to this genome note have been supplied by a Tree of Life collaborator. The Wellcome Sanger Institute employs a process whereby due diligence is carried out proportionate to the nature of the materials themselves, and the circumstances under which they have been/are to be collected and provided for use. The purpose of this is to address and mitigate any potential legal and/or ethical implications of receipt and use of the materials as part of the research project, and to ensure that in doing so, we align with best practice wherever possible. The overarching areas of consideration are:

- Ethical review of provenance and sourcing of the material
- Legality of collection, transfer and use (national and international).

Each transfer of samples is undertaken according to a Research Collaboration Agreement or Material Transfer Agreement entered into by the Tree of Life collaborator, Genome Research Limited (operating as the Wellcome Sanger Institute), and in some circumstances, other Tree of Life collaborators.

## Data Availability

European Nucleotide Archive: Emmelia trabealis. Accession number PRJEB80925. The genome sequence is released openly for reuse. The *Emmelia trabealis* genome sequencing initiative is part of the Sanger Institute Tree of Life Programme (PRJEB43745) and Project Psyche (PRJEB71705). All raw sequence data and the assembly have been deposited in INSDC databases. The genome will be annotated using available RNA-Seq data and presented through Ensembl at the European Bioinformatics Institute. Raw data and assembly accession identifiers are reported in Table 1 and Table 2. Pipelines used for genome assembly at the WSI Tree of Life are available at https://pipelines.tol.sanger.ac.uk/pipelines. Table 5 lists software versions used in this study.

## Author information

Contributors are listed at the following links:

- Members of the Wellcome Sanger Institute Tree of Life Management, Samples and Laboratory team
- Members of Wellcome Sanger Institute Scientific Operations – Sequencing Operations
- Members of the Wellcome Sanger Institute Tree of Life Core Informatics team
- Members of the Tree of Life Core Informatics collective
- Members of the Project Psyche Community
