# Supplementary material for: The genome sequence of the Spotted Sulphur, Emmelia trabealis (Scopoli, 1763) (Lepidoptera: Noctuidae)
Source: Wellcome Open Res. 2026 Feb 16;11:117. [Version 1] doi: 10.12688/wellcomeopenres.25825.1 (PMC13227099; doi:10.12688/wellcomeopenres.25825.1)
Supplement: Peer review history

# Reviewer report: Reviewer response for version 1

**DOI:** 10.21956/wellcomeopenres.28426.r149836

**Published:** 2026-06-01

**Authors:** Annabel Whibley

**Affiliations:** 1 Bragato Research Institute, Clyde, New Zealand

Vila and colleagues report the reference genome assembly of the Spotted Sulphur *Emmelia trabealis* as part of project Psyche. The Data Note follows templated reporting and pipelines for sample collection and processing and data analysis. Public accession links to data and metadata are active. The genome assembly is of excellent quality and comprehensively analysed. The individual is highly heterozygous and both haplotypes are reported. Haplotype 1 has been scaffolded to chromosome level and >98% of the assembly has been assigned to chromosomes. The authors note some large chromosome inversions between the two haplotypes, and I am encouraged to see this information included in the report.

 Minor Comments:

 In the Background section, the phrase  “In other parts of its range” doesn’t make sense. Perhaps “In some parts of ..”?

 I would recommend setting out “asl” in full.

Are sufficient details of methods and materials provided to allow replication by others?

Yes

Is the rationale for creating the dataset(s) clearly described?

Yes

Are the datasets clearly presented in a useable and accessible format?

Yes

Are the protocols appropriate and is the work technically sound?

Yes

Reviewer Expertise:

Bioinformatics, Genomics

I confirm that I have read this submission and believe that I have an appropriate level of expertise to confirm that it is of an acceptable scientific standard.

# Reviewer report: Reviewer response for version 1

**DOI:** 10.21956/wellcomeopenres.28426.r152962

**Published:** 2026-04-28

**Authors:** Jonathan Badger

**Affiliations:** 1 National Cancer Institute, Bethesda, Maryland, USA; 2 National Cancer Institute, Misrata, Libya

The authors have assembled the genome of E. trabealis, a moth with wide distribution across parts of Europe, Northern Africa, and even parts of Asia, from a single female individual. The PacBio sequence data was assembled into two haplotypes and the W and Z sex chromosomes as well as the mitochondrial genome were obtained. Hi-C data was also obtained and is part of the Bioproject, The assembly statistics look good and the data is publicly available through the ENA.

 This is a straightforward Data Note and the authors have followed the standard organization for such an article. I see nothing that needs to be changed.

Are sufficient details of methods and materials provided to allow replication by others?

Yes

Is the rationale for creating the dataset(s) clearly described?

Yes

Are the datasets clearly presented in a useable and accessible format?

Yes

Are the protocols appropriate and is the work technically sound?

Yes

Reviewer Expertise:

I am a computational genomicist, currently working in the biomedical field, but I have also worked on environmental projects in the past and have a currently ongoing side project on the genomes of aphids and their bacterial endosymbionts so I have familiarity with insect genomics.

I confirm that I have read this submission and believe that I have an appropriate level of expertise to confirm that it is of an acceptable scientific standard.
